# Multiple-Factors-Induced Rheumatoid Arthritis Synoviocyte Activation Is Attenuated by the α2-Adrenergic Receptor Agonist Dexmedetomidine

**DOI:** 10.3390/ijms241310756

**Published:** 2023-06-28

**Authors:** Dongun Lee, Jeong Hee Hong

**Affiliations:** Department of Health Sciences and Technology, Lee Gil Ya Cancer and Diabetes Institute, GAIHST, Gachon University, 155 Getbeolro, Yeonsu-gu, Incheon 21999, Republic of Korea; sppotato1@gmail.com

**Keywords:** dexmedetomidine, TNF-α, EGF, IL-6, rheumatoid arthritis, FLS

## Abstract

Dexmedetomidine (Dex) has analgesic and sedative properties and anti-inflammatory functions. Although the effects of Dex on arthritis have been revealed, the physiological mechanism underlying the interaction between Dex and rheumatoid arthritis (RA)-mediated inflammatory cytokines has not been fully studied. Inflamed and migrated fibroblast-like synoviocytes (FLSs) are involved in RA severity. Thus, we aimed to determine the effects of Dex on RA-FLSs treated with inflammatory cytokines and a growth factor as multiple stimulating inputs. TNF-α, IL-6, and EGF as multiple stimulating inputs increased the cAMP concentration of RA-FLSs, while Dex treatment reduced cAMP concentration. Dex reduced electroneutral sodium-bicarbonate cotransporter 1 (NBCn1) expression, NBC activity, and subsequent RA-FLS migration. The mRNA expression levels of RA-related factors, such as inflammatory cytokines and osteoclastogenesis factors, were enhanced by multiple-input treatment. Notably, Dex effectively reduced these expression levels in RA-FLSs. These results indicate that multiple inflammatory or stimulating inputs enhance RA-FLS migration, and treatment with Dex relieves activated RA-FLSs, suggesting that Dex is a potential therapeutic drug for RA.

## 1. Introduction

Rheumatoid arthritis (RA) is a chronic autoimmune disease caused by various factors. Various inflammatory cytokines and growth factors stimulate RA progression by acting as signaling inputs. Synovial fluid of RA contains various inflammatory cytokines, growth factors, and enzymes including metallopeptidase, elastase, and amylase [1,2,3]. These molecules activate RA-fibroblast-like synoviocytes (RA-FLSs) through regulation of proliferation, migration to synovial tissue, and inflammatory reactions [1].

Dexmedetomidine (Dex), which is known as a pain reliever and activator of the α2-adrenergic receptor (α2-AR), attenuates the cAMP level [4,5] and plays an anti-inflammatory role by inhibiting the NF-κB [6,7,8] and Janus kinase 2 (JAK2)-signal transducer and activator of transcription 3 (STAT3) signaling pathways [9,10,11]. Dex has been reported to play an inhibitory role in various inflammation-associated diseases, such as RA, and has thus been reviewed [12]. Mechanistically, Dex inhibits RA-FLS migration by inhibiting the nuclear factor-κB (NF-κB)-associated signaling pathway [13]. In addition, Dex is considered to be an effective strategy with popularity and relatively low price [14,15].

Sodium-bicarbonate cotransporters (NBCs) are involved in various fundamental cellular processes, including pH modulation, sodium influx, and supply of bicarbonate to carbonic anhydrases [16,17]. Previously, we demonstrated that the electroneutral NBC1 (NBCn1) is involved in FLS migration by stimulating RA synovial fluids, including the inflammatory cytokine TNF-α [18]. The activity of NBCs is modulated by several factors, such as cAMP stimulation [19,20] and interaction with the IP_3_ receptor (IP_3_R)-binding protein released with IP_3_ (IRBIT) [21,22]. The interaction between NBCn1 and IRBIT has been evaluated in lung cancer cell migration; IRBIT expression provides membrane stability against NBCn1 [22].

Cellular migration occurs through the launch of various migratory modules, such as ion transporters and water channels [23,24]. Although Dex attenuates inflammatory and migratory effects, whether Dex modulates migratory modules and NBC activity, and its interaction with the regulatory protein IRBIT, remains unclear in RA-FLSs. Thus, we investigated the role of Dex in multiple-signaling-input-mediated inflammation and the modulation of NBC activity and IRBIT in RA-FLSs.

## 2. Results

### 2.1. Dex Inhibited NBC Activities Stimulated by Multiple Inflammatory or Proliferative Inputs in RA-FLSs

**Figure 1:**
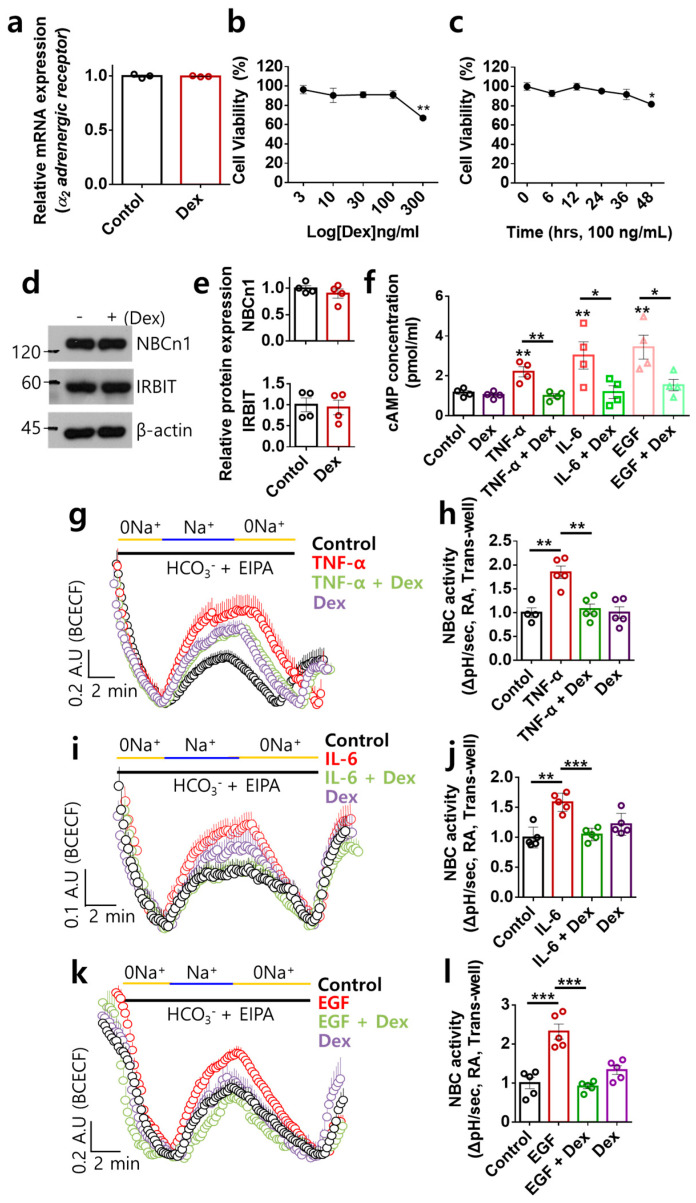
Dex inhibited NBC activities stimulated by multiple inflammatory or proliferative inputs in RA-FLSs. (**a**) mRNA expression of *α2-AR* in human RA-FLS cells with or without Dex treatment. (**b**) Dot plots represent the cellular viability of RA-FLSs following dose-dependent treatment with Dex (n = 3, ** *p* < 0.01). (**c**) Dot plots represent the cellular viability of RA-FLSs following time-dependent treatment with Dex (100 ng/mL, n = 3, * *p* < 0.05). (**d**) Protein expression of NBCn1 and IRBIT in human RA-FLS cells with or without Dex. (**e**) Bars represent the mean ± SEM (n = 3). (**f**) Bars represent the mean ± SEM of cAMP concentration in RA-FLSs with or without Dex treatment (n = 4, * *p* < 0.05, ** *p* < 0.01). (**g**–**l**) NBC activity was assessed by measuring the changes in pH_i_ with 10 ng/mL TNF-α (**g**), 50 ng/mL IL-6 (**i**), and 10 ng/mL EGF (**k**) with or without 100 ng/mL Dex in RA-FLSs for 6 h using transwell assay. Bars represent the mean ± SEM of TNF-α (**h**), IL-6 (**j**), and EGF (**l**) (n = 5, ** *p* < 0.01 and *** *p* < 0.001).

We determined whether Dex modulated the *α2-AR* expression level in RA-FLSs. Stimulation of RA-FLSs with Dex did not change the *α2-AR* expression level (Figure 1a). To determine the optimal conditions for Dex exposure, cell viability was measured in a dose- and time-dependent manner. Dex stimulation was performed at 100 ng/mL for 24 h (Figure 1b,c). Previously, we revealed that the expression of NBCn1 is mediated by the inflammatory cytokine TNF-α and has a migratory role in RA-FLSs [18]. IRBIT is known to modulate NBCs [21,22]. We determined whether α2-AR stimulation with Dex modulated the expression of NBCn1 and IRBIT in RA-FLSs. Dex stimulation did not modulate the expression of NBCn1 or IRBIT (Figure 1d,e). The inflamed synovium contains various inflammatory cytokines and growth factors [1,25]; thus, we speculated that RA-FLSs are exposed to multiple inflammatory inputs. To determine the role of Dex in NBC activity stimulated by multiple cytokines or proliferative factors through α2-AR activation, we stimulated cells with inflammatory cytokines (TNF-α and IL-6) and the proliferative factor EGF. Therefore, we hypothesized that NBCn1 is a single migratory target that responds to multiple inputs. Dex is known to exert an inhibitory effect on cAMP [5,26]. In this study, we measured the changes in cAMP level based on treatment with TNF-α, IL-6, or EGF, as multiple inputs, with or without Dex. Dex inhibited multiple-stimulation-induced cAMP levels (Figure 1f). Enhanced cAMP levels are associated with inhibitory effects on NBC activity [20]. Thus, we hypothesized that Dex determines whether Dex-mediated cAMP inhibition enhances NBC activity. NBC activity was stimulated by treatment with multiple inputs (Figure 1g–l). Dex attenuated TNF-α-, IL-6-, or EGF-stimulated NBC activities (Figure 1h,j,l). Furthermore, multiple-input-mediated enhanced NBC activity was independent of cAMP levels. Overall, Dex effectively inhibited NBC activities stimulated by multiple inflammatory or proliferative inputs in RA-FLSs.

### 2.2. Multiple-Input-Mediated RA-FLS Activation Is Not Associated with Akt, Erk, and NF-κB Signaling

**Figure 2:**
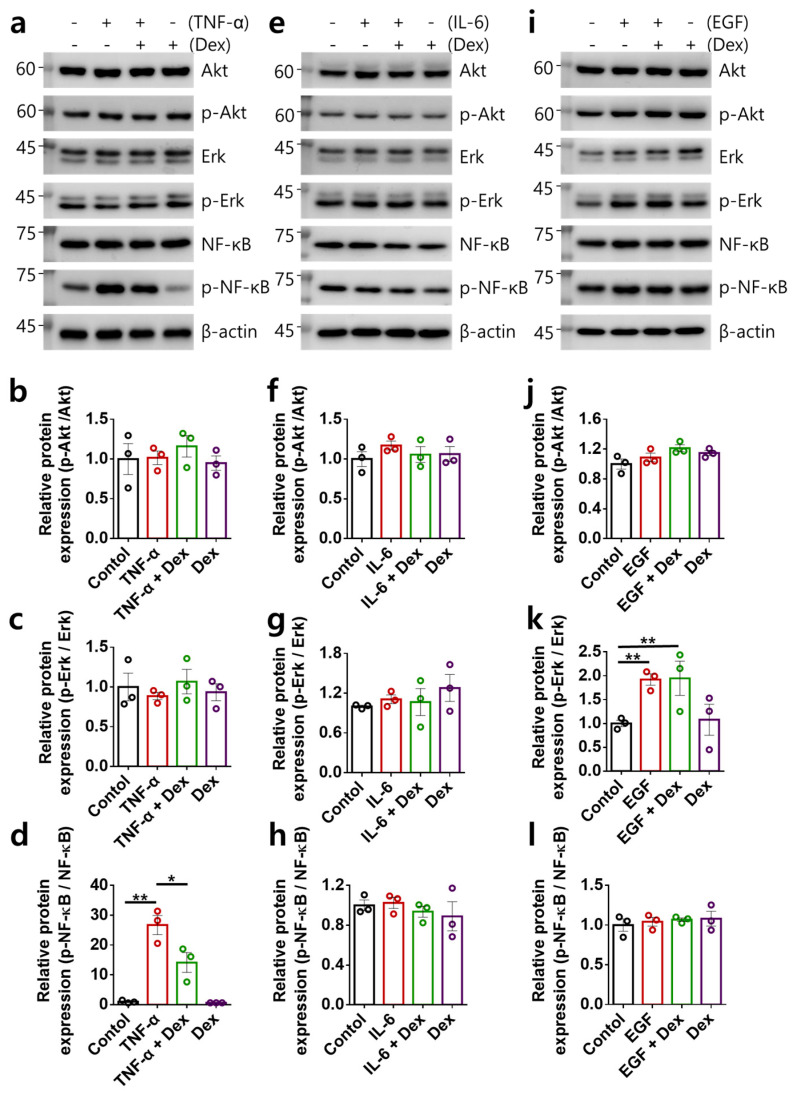
Inhibitory role of Dex in the intracellular signaling pathway in RA-FLSs. (**a**) Protein expression of Akt, Erk, and NF-κB (total and phosphorylated forms) in human RA-FLS cells with or without 10 ng/mL TNF-α and 100 ng/mL Dex. (**b**–**d**) Bars represent the mean ± SEM of p-Akt (**b**), p-Erk (**c**), and p-NF-κB (**d**) protein expression (n = 3, * *p* < 0.05, ** *p* < 0.01). (**e**) Protein expression of Akt, Erk, and NF-κB (total and phosphorylated forms) in human RA-FLS cells with or without 50 ng/mL IL-6 and 100 ng/mL Dex. (**f**–**h**) Bars represent the mean ± SEM of p-Akt (**f**), p-Erk (**g**), and p-NF-κB (**h**) protein expression. (**i**) Protein expression of Akt, Erk, and NF-κB (total and phosphorylated forms) in human RA-FLS cells with or without 10 ng/mL EGF and 100 ng/mL Dex. (**j**–**l**) Bars represent the mean ± SEM of p-Akt (**j**), p-Erk (**k**), and p-NF-κB (**l**) protein expression (n = 3, ** *p* < 0.01).

We verified the mechanical role of Dex in intracellular Akt, ERK, and NF-κB signaling in RA-FLSs [27,28,29]. We determined the levels of intracellular signaling proteins in multiple-input-stimulated RA-FLSs (Figure 2a–l). The levels of Akt and Erk were not changed by treatment with TNF-α; however, the phosphorylation of NF-κB was increased by TNF-α stimulation (Figure 2a–d). Although treatment with Dex alone did not modulate the protein expression of Akt, Erk, and NF-κB, the increased phosphorylation of NF-κB by TNF-α treatment was attenuated by Dex (Figure 2a–d). In contrast, IL-6 stimulation did not alter the phosphorylation of signaling proteins (Figure 2e–h). Treatment with EGF increased the phosphorylation of Erk; however, Dex did not attenuate this increase (Figure 2i,k). In addition, the levels of Akt and NF-κB were not changed by treatment with EGF (Figure 2i,j,l). Although treatment with TNF-α or EGF caused changes in NF-κB or Erk, the multiple inputs did not correlate with Akt, Erk, and NF-κB signaling. These results indicate that RA-FLS activation mediated by multiple inputs is not associated with Akt, Erk, and NF-κB signaling. 

### 2.3. Dex Attenuates RA-FLS Migration Stimulated by Multiple Inputs

**Figure 3:**
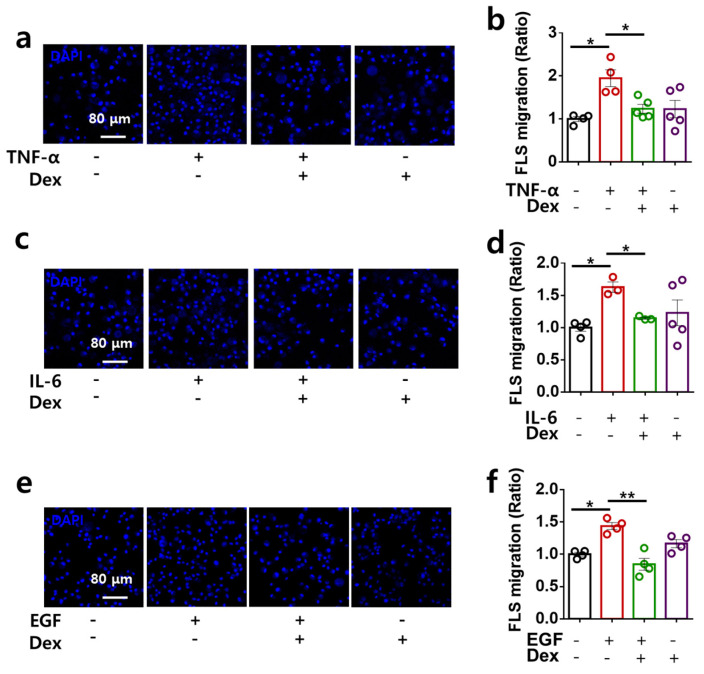
Dex treatment attenuated the migration of RA-FLSs stimulated by multiple inputs. RA-FLS migration was measured following stimulation with 10 ng/mL TNF-α (**a**), 50 ng/mL IL-6 (**c**), and 10 ng/mL EGF (**e**) in the transwell culture system with or without 100 ng/mL Dex. Analysis of the total DAPI intensity for RA-FLS migration with TNF-α (**b**), IL-6 (**d**), and EGF (**f**). Bars represent the mean ± SEM (n = 5, * *p* < 0.05 and ** *p* < 0.01).

Multiple inputs enhance NBC activity, and activation of the migratory module, NBCn1, induces RA-FLS migration [18]. Therefore, we determined the functional inhibitory role of Dex in cellular migration in multiple-input-stimulated RA-FLSs. Transwell migration assays revealed that multiple inputs enhanced cellular migration, whereas co-stimulation with Dex reduced the migratory effect of the multiple inputs (Figure 3a–f). Thus, Dex attenuated multiple stimulations with inflammatory cytokines and growth factor-mediated RA-FLS migration.

### 2.4. Directional Stimulation of TNF-α- and EGF-Mediated Membranous NBCn1 Expression in RAFLS Is Attenuated by Dex 

**Figure 4:**
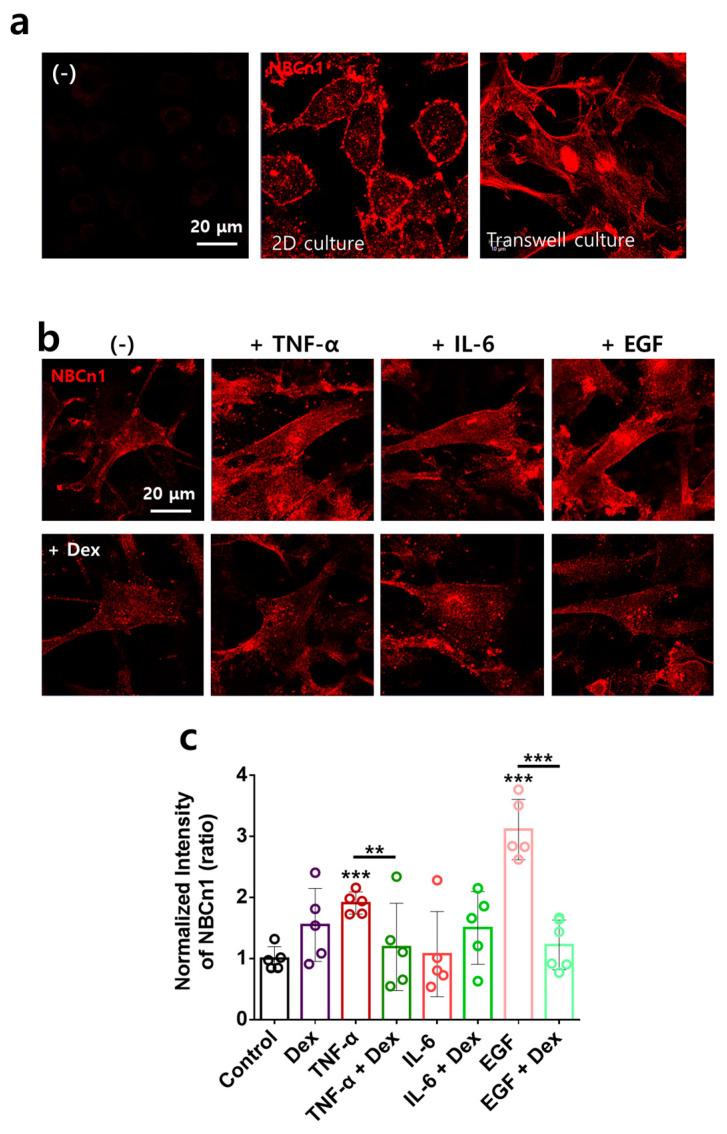
Directional stimulation of TNF-α- and EGF-mediated membranous NBCn1 expression in RA-FLSs was attenuated by Dex. (**a**) Immunofluorescence staining of NBCn1 (red) using 2D culture on coverslips or the transwell membrane. Left images (−) indicate the negative control (absence of NBCn1 antibodies). Scale bar represents 20 μm. (**b**) Immunofluorescence staining of NBCn1 (red) using transwell membrane after treatment with 10 ng/mL TNF-α, 50 ng/mL IL-6, and 10 ng/mL EGF on the bottom plate with or without 100 ng/mL Dex in the upper chamber for 6 h. Scale bar represents 20 μm. (**c**) Bars represent the mean ± SEM of the normalized band intensity of NBCn1 in RA-FLSs (n = 5, ** *p* < 0.01 and *** *p* < 0.001).

NBCn1 is known as a plasma membrane-associated transporter. To determine the membrane localization of NBCn1, RA-FLSs were immunostained with NBCn1 in 2D and transwell culture systems. NBCn1 was expressed in the cellular membranes of both culture systems (Figure 4a). NBCn1 in transwell-cultured RA-FLSs displayed a steeper and more unified localization than that in 2D-cultured RA-FLSs (Figure 4a). Thus, we performed experiments to determine the migratory modules in the transwell culture system. RA-FLSs were examined to determine NBCn1 expression in the presence of multiple inputs. Membranous NBCn1 expression was enhanced by TNF-α and EGF stimulation but not by IL-6 (Figure 4b,c). Dex attenuated the TNF-α and EGF stimulation-mediated membranous NBCn1 expression (Figure 4b,c). These results indicate that Dex attenuated TNF-α- and EGF-stimulated membranous NBCn1 expression in RA-FLSs.

### 2.5. TNF-α- and EGF-Mediated NBCn1 Expression Is Attenuated by Dex with No Change in IRBIT in RA-FLSs

**Figure 5:**
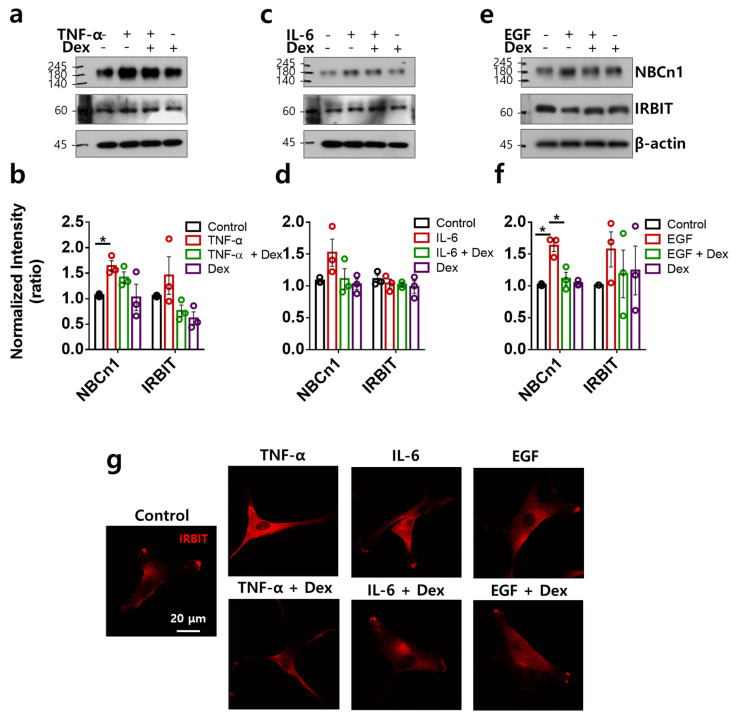
TNF-α- and EGF-mediated NBCn1 expression was attenuated by Dex with no change in IRBIT in RA-FLSs. (**a**–**f**) Protein expression levels of NBCn1 and IRBIT following treatment with 10 ng/mL TNF-α (**a**), 50 ng/mL IL-6 (**c**), and 10 ng/mL EGF (**e**) with or without Dex in RA-FLSs for 6 h using a transwell membrane. β-actin was used as a loading control. Bars represent the mean  ±  SEM of the normalized band intensity of NBCn1 and IRBIT following treatment with 10 ng/mL TNF-α (**b**), 50 ng/mL IL-6 (**d**), and 10 ng/mL EGF (**f**) with or without Dex in RA-FLSs (n = 3, * *p* < 0.05). (**g**) Immunofluorescence staining of IRBIT (red) using a transwell membrane after treatment with 10 ng/mL TNF-α, 50 ng/mL IL-6, and 10 ng/mL EGF on the bottom plate with or without 100 ng/mL Dex in the upper chamber for 6 h. Scale bar represents 20 μm.

IRBIT is broadly expressed and coordinates the regulation of ion channels and transporters [30,31]. We determined the expression of NBCn1 and its modulatory factor, IRBIT. TNF-α and EGF stimulated NBCn1 expression, whereas IRBIT expression did not change in RA-FLSs (Figure 5a,b,e,f). IL-6 mildly enhanced NBCn1 expression; however, the difference was not statistically significant (Figure 5c,d). Dex attenuated the TNF-α and EGF stimulation-mediated NBCn1 expression (Figure 5a–f). The expression of IRBIT protein was mildly enhanced in RA-FLSs stimulated with TNF-α and EGF; however, no statistical change was found. IRBIT expression was not modulated by multiple inputs, with or without Dex (Figure 5a–f). IRBIT, which is a binding component of IP_3_R, is released by IP_3_ binding from IP_3_R [30,31]. IRBIT localization revealed cytosolic expression and aggregate formation at the edge of the membrane with or without multiple inputs and Dex (Figure 5g). These results indicate that Dex did not modulate multiple-input-stimulated IRBIT expression.

### 2.6. Dex Treatment Inhibits Multiple RA-Associated Factors in RA-FLSs

**Figure 6:**
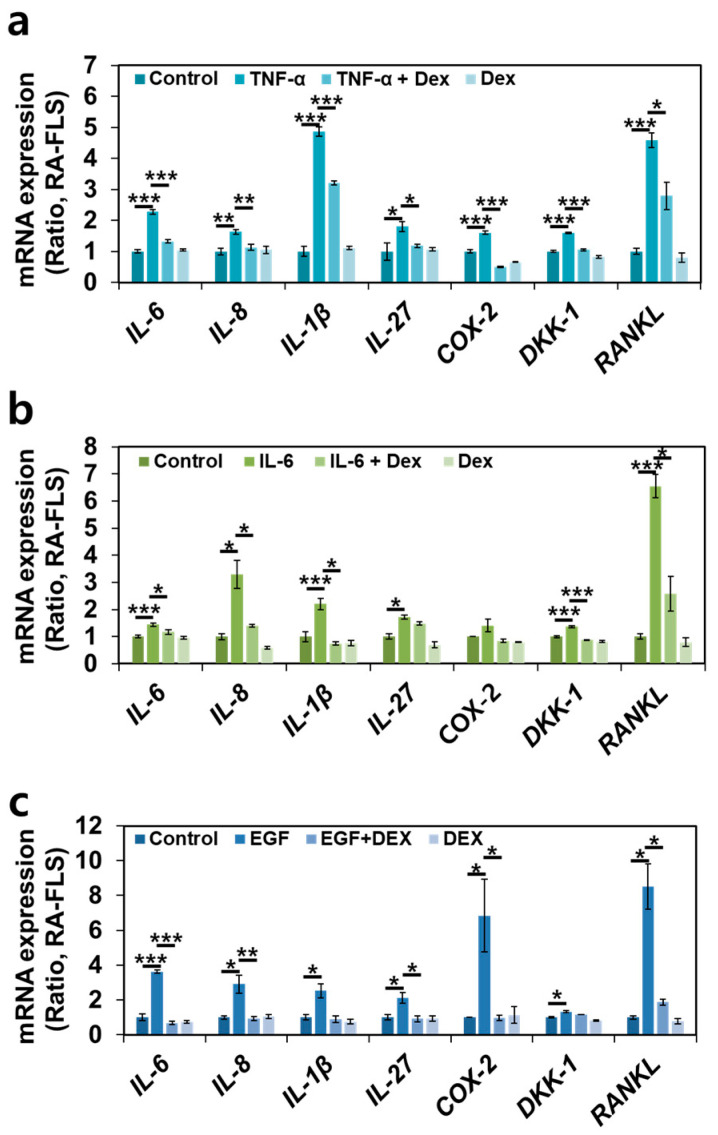
Dex inhibited multiple-input-associated RA factors in RA-FLSs. The relative mRNA levels of *IL-6*, *IL-8*, *IL-1β*, *IL-27*, *COX-2*, *DKK-1*, and *RANKL* stimulated with 10 ng/mL TNF-α (**a**), 50 ng/mL IL-6 (**b**), and 10 ng/mL EGF (**c**) with or without 100 ng/mL Dex for 48 h in transwell-cultured RA-FLSs. Bars represent mean ± SEM (n = 4, * *p* < 0.05, ** *p* < 0.01, and *** *p* < 0.001).

Owing to the inhibitory effect of Dex on RA-FLS migration, we determined the inhibitory effect of Dex on RA-associated factors to verify its therapeutic endpoint. Various RA-associated inflammatory factors, such as *IL-6*, *IL-8*, *IL-1β*, *IL-27*, and *COX-2* [18,32], RA-associated joint remodeling factor *Dickkopf-1* (*DKK-1*) [33], and osteoclastogenesis factor *receptor activator of nuclear factors κB ligand* (*RANKL*) [34] in multiple-input-stimulated RA-FLSs with or without Dex were evaluated in a transwell culture system. The multiple inputs were found to stimulate RA-associated inflammatory and joint factors, whereas Dex attenuated the enhanced mRNA expression of these factors (Figure 6a–c). These results indicate that Dex plays an inhibitory role in inflammatory cytokine- and bone damage-associated mRNA expression.

## 3. Discussion

**Figure 7:**
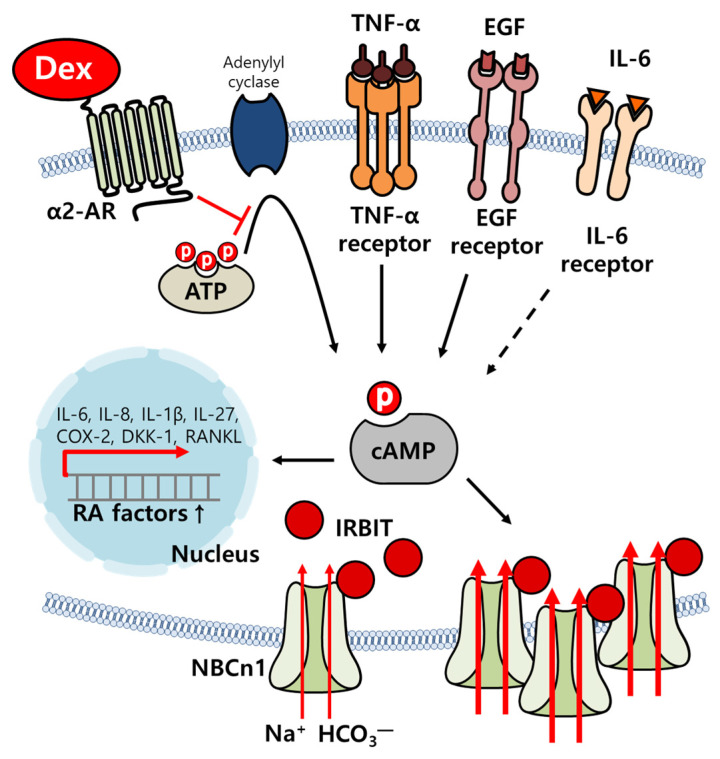
Schematic illustration of RA-FLS activation by multiple inputs and the inhibitory role of Dex. Multiple-input-mediated cAMP stimulation increased NBCn1 expression and activity by upregulating RA factors, such as *IL-6*, *IL-8*, *IL-1β*, *IL-27*, *COX-2*, *DKK-1*, and *RANKL*, which were attenuated by Dex treatment. Dex: dexmedetomidine, AR: adrenergic receptor, IL: interleukin, COX-2: cyclooxygenase-2, *DKK-1: Dickkopf-1*, *RANKL: receptor activator of nuclear factors κB ligand*, IRBIT: the IP_3_ receptor-binding protein released with IP_3_, NBCn1: electroneutral sodium-bicarbonate cotransporter 1.

The inflammatory process in RA occurs owing to the release of inflammatory cytokines in the synovium. Difficulties in the treatment of RA are caused by multiple targets that attenuate inflammatory cytokines. Dex is known to induce sedation and protect against inflammatory processes in various tissues, such as the brain, heart, and kidney [35,36,37,38]. In this study, Dex attenuated the inflammatory effects of cellular responses mediated by multiple inflammatory inputs, including cellular migration and cytokine production in RA-FLSs (Figure 7). Moreover, inflammatory input-mediated NBC activity, which is involved in migration, was attenuated by treatment with Dex.

Multiple-input-mediated bicarbonate transporter activity is another target of input-associated inflammatory signaling. Our previous study revealed that the recruitment of NBCn1 by inflammatory synovial fluid from patients with RA provides a new strategy for RA treatment [18]. NBCn1 is encoded by the solute carrier (SLC)4 transporter family, which plays an essential physiological role in cellular acid–base homeostasis [39,40]. The SLC4 transporter family is involved in several human diseases, including renal tubular acidosis, ocular abnormalities, migration, blindness, auditory impairment, and heart failure [41,42,43]. In addition to the physiological role of NBCn1 in bicarbonate transport, we addressed a new paradigm of RA treatment through NBCn1. In this study, we evaluated the multiple stimulating inputs that modulate NBC activity. Multiple stimulating input-associated NBC as a signaling endpoint can be a therapeutic target for RA by attenuating multiple-input-mediated signaling. Dex was found to relieve multiple-input-mediated NBC activation, suggesting that Dex could be a therapeutic drug for RA. 

## 4. Materials and Methods

### 4.1. RA-FLS Culture

Human RA-FLSs, originating from RA synovial tissues, were purchased from Cell Applications, Inc. (San Diego, CA, USA). RA-FLSs were maintained in Dulbecco’s Modified Eagle’s medium (DMEM, Invitrogen, Waltham, MA, USA) containing 10% fetal bovine serum (FBS; Invitrogen) and 100 U/mL penicillin–streptomycin (Invitrogen) and incubated at 37 °C in 5% CO_2_/95% air. When the RA-FLSs reached 80% cell density, the cell culture medium was aspirated, and the RA-FLSs were washed with Dulbecco’s phosphate-buffered saline (DPBS, Welgene, Gyeongsan, Republic of Korea), treated with cell detachment solution trypsin/ethylenediaminetetraacetic acid (EDTA, Invitrogen) for 1 min, and cultured until use. RA-FLS cells were starved by using serum-free media for 24 h before stimulation of EGF. RA-FLSs were used within 10 passages in all experiments.

### 4.2. Total RNA Extraction and Quantitative Real-Time Polymerase Chain Reaction (RT-qPCR)

Total RNA was isolated from primary RA-FLSs cultured in a transwell system using Ribo^Ex^ (GeneAll, Songpa, Gyeongsan, Republic of Korea). Ribo^Ex^ was added to RA-FLSs, and the cells were broken via sonication. Following the addition of chloroform, the sample was centrifuged at 12,000× *g* for 15 min at 4 °C. The supernatant was placed in a new tube, isopropanol was added to the tube, and the mixture was incubated on ice for 10 min. The mixture was centrifuged at 12,000× *g* for 10 min at 4 °C. Thereafter, the supernatant was removed and washed with 70% ethanol. After centrifugation at 7500× *g* for 10 min at 4 °C, the supernatant was removed and dried in air to dissolve the pellets in DEPC-treated water. RNA was quantified using a Spectrophotometer ND-1000 (Thermo Fisher, Waltham, MA, USA) and then amplified using a cDNA synthesis kit from Enzynomics (Daejeon, Republic of Korea), according to the manufacturer’s instructions. RT-qPCR was performed using PowerUp SYBR Green Master Mix (Applied Biosystems, Waltham, MA, USA), primers, and the QuantStudio 3 RT-PCR system (Applied Biosystems). The primers used in this study are listed in Table 1. The following RT-qPCR cycling protocol was employed: UDG activation at 50 °C for 2 min, Dual-Lock DNA polymerase at 95 °C for 2 min, denaturation at 95 °C for 15 s, annealing at 55 °C for 15 s, and extension at 72 °C for 1 min.

### 4.3. MTT Assay

FLSs (~5000 cells) were cultured in 96-well plates for 24 h after the addition of the indicated reagents and medium for 48 h with 100 ng/mL Dex. The cells were treated with 100 ng/mL Dex for 6, 12, 24, 36, and 48 h. Tetrazolium bromide (2 mg; MTT, Merck, Burlington, MA, USA) dye was mixed with 1 mL of PBS, and the cells were treated with 100 μL of MTT dye and incubated for 2 h in the dark. The incubation medium was carefully aspirated from the plates. A total of 100 µL of DMSO was added to the plates, and the absorbance was measured at 570 nm using a fluorescence microplate reader (VICTOR X3, PerkinElmer, Waltham, MA, USA). 

### 4.4. Western Blot

FLSs were cultured in a culture dish (termed monoculture) and transwell insert dish (termed transwell culture) with 10 ng/mL TNF-α, 50 ng/mL IL-6, 10 ng/mL EGF, and 100 ng/mL Dex for 6 h. RA-FLS lysates were prepared in lysis buffer containing 20 mM Tris, 150 mM NaCl, 2 mM EDTA, 1% Triton X-100, and a protease inhibitor mixture and were treated as previously described [44]. Briefly, cellular proteins were denatured in SDS sample buffer at 37 °C for 30 min. Denatured protein samples (30 μg) were subjected to sodium dodecyl sulfate-polyacrylamide gel electrophoresis. The proteins were visualized with NBCn1, IRBIT, Akt, phosphorylated form (p)-Akt, Erk, p-Erk, NF-κB, p-NF-κB, and β-actin antibodies using enhanced luminescence solution (Thermo Scientific) and developed on X-ray film (Kodak, New York, NY, USA).

### 4.5. Measurement of Cyclic Adenosine Monophosphate (cAMP)

FLSs were monocultured with 10 ng/mL TNF-α, 50 ng/mL IL-6, 10 ng/mL EGF, and 100 ng/mL Dex for 6 h. The cAMP concentration was measured using a cAMP ELISA kit (Cayman Chemical, Ann Arbor, MI, USA) according to the manufacturer’s instructions. Briefly, cultured RA-FLSs were lysed using lysis buffer and mixed with tracer and antiserum solutions. The mixture was added to a mouse anti-rabbit IgG-coated 96-well plate, and the samples were incubated at 4 °C for 18 h. After incubation, the wells were washed with wash buffer, and Ellman’s reagent was added to each well. The plates were incubated at room temperature for 120 min in the dark. The absorbance of each sample was measured at 415 nm, and the concentration of cAMP was calculated using the standard curve in Supplementary Figure S1.

### 4.6. Measurement of the Na^+^-HCO_3_^−^ Cotransporter (NBC) Activity

For pH_i_ measurement-based NBC activity, changes in the pH_i_ of RA-FLSs were determined using 2′-7′-bis-(carboxyethyl)-5-(and-6)-carboxyfluorescein (BCECF-AM; Teflabs, Austin, TX, USA) at dual excitation wavelengths of 440 and 495 nm and the single emission wavelength of 530 nm. RA-FLSs attached to coverslips were loaded into the chamber with 6 µM BCECF-AM in the presence of 0.05% Pluronic F-127 for 15 min at room temperature. The dye-loaded RA-FLSs were perfused with a physiological salt solution (the composition is presented elsewhere [45]) for at least 5 min to stabilize the fluorescence before measuring pH_i_ at 37 °C. The NBC activity of RA-FLSs was measured by incubating the cells with CO_2_-saturated HCO_3_^−^-buffered media with 5-(N-ethyl-N-isopropyl) amiloride (EIPA) (Sigma, Saint Louis, MO, USA) and the indicated reagents, including 100 ng/mL Dex (dexmedetomidine hydrochloride; Sigma, Saint Louis, MO, USA), 10 ng/mL TNF-α (R&D Systems, Minneapolis, MN, USA), 10 ng/mL EGF (Thermo Fisher, Waltham, MA, USA), and 50 ng/mL IL-6 (PeproTech, Cranbury, NJ, USA), followed by acidification with Na^+^-free HCO_3_^−^-buffered media. The emitted fluorescence of the dye-loaded cells was monitored using a CCD camera (Retiga 6000, Q-imaging, Tucson, AZ, USA) attached to an inverted microscope (Olympus, Tokyo, Japan) and analyzed using a MetaFluor system (Molecular Devices, San Jose, CA, USA). All BCECF fluorescence images were obtained at 1 s intervals, and the background fluorescence of the image was subtracted from the raw background signals at each wavelength. NBC activity was determined from the derivatives of the initially enhanced slopes from the first 1 min of pH_i_ increase in Na^+^-free HCO_3_^−^-buffered media, as previously reported [18,46].

### 4.7. Transwell Membrane-Based Migration Assay 

The RA-FLS migration assay was conducted using a transwell polycarbonate membrane (6.5 mm insert, 8.0 μm pore size). The inserted membranes were filled with 200 μL of RA-FLSs (5 × 10^4^ cells; Cell Applications, Inc., San Diego, CA, USA) containing 1% FBS, reagents, and 100 ng/mL Dex. The bottom plate was treated with 10 ng/mL TNF-α, 10 ng/mL EGF, and 50 ng/mL IL-6 dissolved in 500 μL of DMEM, and incubated for 6 h. DMEM on the bottom plate was removed, and chilled methanol was added carefully. The inserted membranes were then soaked for 1 min at −20 °C. The chilled methanol was removed, and the inserted membranes were washed three times with phosphate-buffered saline (PBS). The DAPI solution was mixed with distilled water and loaded onto the bottom plate. The plates were then incubated for 30 min in the dark. The medium was carefully removed from the insert. Distilled water (DW) was added to the bottom plate at room temperature, and DAPI fluorescence of the inserted membrane was measured at 405 nm using an LSM 700 Zeiss confocal microscope (Carl Zeiss, Jena, Germany). RA-FLS migration was determined based on the number of nuclei stained with DAPI on the transwell membrane. 

### 4.8. Immunostaining of the Inserted Membrane in the Transwell Culture System

After the migration assay, the inserts were restored for immunostaining. The membrane of the insert was cut, isolated, and immunostained with NBCn1 antibody (Abcam, Cambridge, UK). The isolated membrane was added to a 50 mM cold glycine solution for 10 min at 4 °C, and then a 5% blocking solution was added for 1 h at room temperature in the dark. The antibodies were diluted to 1:100 and incubated overnight at 4 °C. The secondary antibody (rhodamine-tagged goat anti-rabbit IgG) was loaded for 1 h at room temperature. Finally, the membrane was carefully attached and mounted on glass slides with DAPI-containing fluoromount-G (Electron Microscopy Sciences, Hatfield, PA, USA). Fluorescence images were obtained using an LSM 700 Zeiss confocal microscope and analyzed using ZEN software (version 8.1). All imaging data were acquired at the Cell to In Vivo Imaging Core Facility Research Center (CII, Lee Gil Ya Cancer and Diabetes Institute, Gachon University, Incheon, Republic of Korea).

### 4.9. Statistical Analysis

Based on the standard statistical method, the results are expressed as the mean ± standard error of the mean (SEM). Statistical differences between the mean values from the two sample groups were analyzed using Student’s *t*-test. Significance levels were determined using analysis of variance for each experiment (* *p* < 0.05, ** *p* < 0.01, *** *p* < 0.001).

## 5. Conclusions

The RA synovium contains various inflammatory components, such as IL-1β, IL-6, IL-8, IL-17, and TNF-α [47,48,49,50], growth factor EGF [51], and bone remodeling components, such as RANKL [1,52] and DKK [1,53]. Immune cell-mediated inflammatory signals activate RA-FLS aggressiveness and subsequent bone destructive process [1]. Biological disease-modifying anti-rheumatic drugs have been developed to reduce inflammatory components [54,55]. Although vigorous studies have been performed for many years, non-responsive endpoints, limited clinical use, or ethical limitation require a new paradigm for the treatment of RA. For instance, Rzhepakovsky et al. addressed chicken embryo tissue hydrolysate as a potential nutraceutical anti-inflammatory agent and revealed the reduced inflammation of periarticular soft tissues and degeneration of cartilage in a rat arthritis model [56,57]. They addressed the biological protein source, which is an inexpensive, ethical, and epidemiologically safe component, for arthritis treatment. Although its inhibitory role in multiple inflammations needs to be defined, the modulation of multiple-stimulation-input-associated inflammatory signaling by Dex or nutraceutical compounds in arthritis may be a therapeutic candidate for RA. 

## Figures and Tables

**Table 1:** List of primer sequences.

Genes	Sequences (5′→3′)
Human *GAPDH*	(Forward) GAC CTG ACC TGC CGT CTA GAA A (Reverse) CCT GCT TCA CCA CCT TCT TGA
Human *α2-AR*	(Forward) CCC GGT CAT CTA CAC CAT CTT (Reverse) CCC GAC AGA GGA TCT TCT TGA
Human *IL-6*	(Forward) CCC CCA GGA GAA GAT TCC AA (Reverse) CCG TCG AGG ATG TAC CGA ATT
Human *IL-8*	(Forward) GTG GCT GAA CCA GAG TTG GAA (Reverse) TGG TGC ACT GGA GCT GCT T
Human *IL-1β*	(Forward) CCA CGG CCA CAT TTG GTT (Reverse) AGG GAA GCG GTT GCT CAT C
Human *IL-27*	(Forward) GCT TGG ATG TCC CGA AAC C (Reverse) CGC CAC CCC TTG CTA AAA T
Human *COX-2*	(Forward) GCT CAG CCA TAC AGC AAA TCC (Reverse) GTC CGG GTA CAA TCG CAC TT
Human *DKK-1*	(Forward) TGG AAC TCC CCT GTG ATT GC (Reverse) TGG AAC TCC CCT GTG ATT GC
Human *RANKL*	(Forward) GGG TCT TTG TCG CGA TGG TA (Reverse) CTG GTA CTT ATT CCC GCC CG

Abbreviations: *GAPDH*: *glyceraldehyde 3-phosphate dehydrogenase*; *IL-6/8/1β/27*: *interleukin-6/8/1β/27*; *COX-2*: *cyclooxygenase-2*; *DKK-1*: *Dickkopf-1*; *RANKL*: *receptor activator of nuclear factor κB ligand*.

## Data Availability

Not applicable.

## Supplementary Materials

The following supporting information can be downloaded at: https://www.mdpi.com/article/10.3390/ijms241310756/s1.
