# Supplementary material for: Multiple-Factors-Induced Rheumatoid Arthritis Synoviocyte Activation Is Attenuated by the α2-Adrenergic Receptor Agonist Dexmedetomidine
Source: Int J Mol Sci. 2023 Jun 28;24(13):10756. doi: 10.3390/ijms241310756 (PMC10341941; doi:10.3390/ijms241310756)
Supplement: Supplementary file 1 [file ijms-24-10756-s001.zip › ijms-2454694-supplementary.pdf]

Multiple-Factors-Induced Rheumatoid Arthritis Synoviocyte Activation Is Attenuated by the α2-Adrenergic Receptor Agonist Dexmedetomidine

Dongun Lee and Jeong Hee Hong *

Department of Health Sciences and Technology, Lee Gil Ya Cancer and Diabetes Institute, GAIHST, Gachon University, 155 Getbeolro, Yeonsu-gu, Incheon 21999, Republic of Korea; sppotato1@gmail.com

* Correspondence: minicleo@gachon.ac.kr; Tel.: +82-32-899-6682

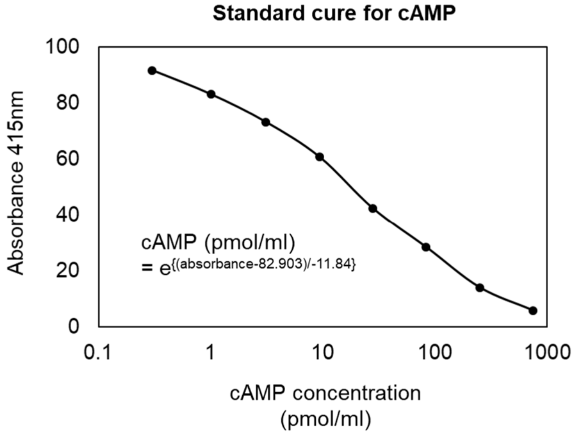

Supplementary Figure S1. Standard curve for cAMP measurement.

The standard curve for calculation of cAMP concentration by using cAMP ELISA kit.
